# Improved Yield Prediction of Winter Wheat Using a Novel Two-Dimensional Deep Regression Neural Network Trained via Remote Sensing †

**DOI:** 10.3390/s23010489

**Published:** 2023-01-02

**Authors:** Giorgio Morales, John W. Sheppard, Paul B. Hegedus, Bruce D. Maxwell

**Affiliations:** 1 Gianforte School of Computing, Montana State University, Bozeman, MT 59717, USA; 2 Department of Land Resources and Environmental Sciences, Montana State University, Bozeman, MT 59717, USA

**Keywords:** yield prediction, deep regression, convolutional neural networks, precision agriculture

## Abstract

In recent years, the use of remotely sensed and on-ground observations of crop fields, in conjunction with machine learning techniques, has led to highly accurate crop yield estimations. In this work, we propose to further improve the yield prediction task by using Convolutional Neural Networks (CNNs) given their unique ability to exploit the spatial information of small regions of the field. We present a novel CNN architecture called Hyper3DNetReg that takes in a multi-channel input raster and, unlike previous approaches, outputs a two-dimensional raster, where each output pixel represents the predicted yield value of the corresponding input pixel. Our proposed method then generates a yield prediction map by aggregating the overlapping yield prediction patches obtained throughout the field. Our data consist of a set of eight rasterized remotely-sensed features: nitrogen rate applied, precipitation, slope, elevation, topographic position index (TPI), aspect, and two radar backscatter coefficients acquired from the Sentinel-1 satellites. We use data collected during the early stage of the winter wheat growing season (March) to predict yield values during the harvest season (August). We present leave-one-out cross-validation experiments for rain-fed winter wheat over four fields and show that our proposed methodology produces better predictions than five compared methods, including Bayesian multiple linear regression, standard multiple linear regression, random forest, an ensemble of feedforward networks using AdaBoost, a stacked autoencoder, and two other CNN architectures.

## 1. Introduction

Precision Agriculture (PA) is a crop management technique that uses Information Technologies (ITs) to obtain spatial and temporal information from fields to inform management decisions [1]. The adoption of PA in the last few decades increased awareness of variation in soil and crop conditions, which are important factors when deciding optimal site-specific treatments [2]. The promise of PA is the creation of strategies to make efficient use of the available farming resources (e.g., water, nutrients, and pesticides), avoid waste, minimize environmental impact, and maximize profit [3,4,5]. To achieve this, models are developed that relate input covariate factors, obtained from a variety of sensors and technified machinery, and outcome response variables, such as crop yield. Then simulations are used to predict outcome variables across rates of spatially variable input factors to inform management decisions. On-Farm Precision Experimentation (OFPE), which generates site-specific data on responses to field management, is crucial to developing these models and simulations [6,7].

Recently, PA has benefited from the confluence of the growing availability of sensors that can accurately and continuously collect information about fields [8,9], the boom of machine learning, and the development of accessible and fast computational resources [10,11]. In this context, one of the most beneficial areas of PA is crop yield prediction, which is important for national food security as it allows the preparation of import/export policies, estimation of prices, and direct influence on farmer profits [12]. Crop yield prediction provides farmers with tools to make informed decisions, such as designing accurate marketing plans for their products [12] or determining the nitrogen fertilizer rates (N) needed to maximize farmer net return [7,13]. However, the adoption of PA has generally been limited to the use of GPS for autosteering equipment, while the adoption of variably applied inputs, such as nitrogen fertilizer, has been limited to around 10% of adopters [14]. Increased adoption of PA management that improves the efficiency of nitrogen fertilizer use will require accurate methods of crop yield predictions to show farmers the benefits of within-field variable rate applications.

The crop yield prediction task can be automated by using machine learning-based approaches. They generate computational models that attempt to approximate the behavior of the field based on the observed relationships between multi-source covariate factors and crop yield. Some approaches use remotely sensed data as covariate factors, such as satellite imagery captured by the Moderate Resolution Imaging Spectroradiometer (MODIS) and Sentinel missions [15,16,17]. Other approaches incorporate on-ground data; that is, data acquired directly from the field, such as soil electroconductivity or nitrogen rate [18,19]. Others recognize that, to increase the adoption of decision support systems that predict yield, the data used to train machine learning approaches should be limited to those data that are available at a low cost to the farmer, such as from open-source satellite imagery or data gathered from farmer’s equipment. Regardless of the data selected as explanatory variables, machine learning-based approaches pose the yield prediction problem as a regression task. As such, regression models are trained to estimate the crop yield response in terms of production, for example, bushels per acre (bu/ac), as accurately as possible. By doing so, the output model (estimated yield) is treated as an implicit function of the input variables whose functional form is learned based on observed data. Previous works have proposed the use of regression models that process the information of each point of the field and (possibly) from its surroundings to estimate its corresponding yield value. Here, a point of the field refers to a small georeferenced region (e.g., a point may represent an area of 10×10 m).

Yield values of neighboring points in the field are mutually dependent. As a consequence, it is natural to consider a crop yield prediction model that analyzes spatial neighborhoods of points [20]. What is more, it is feasible to consider a prediction model whose outputs correspond to the predicted yield values of all the points within these neighborhoods, not only to the central point of the neighborhood. Given that we are considering the use of models with two-dimensional (2-D) inputs and 2-D outputs, we refer to this problem as a two-dimensional regression. Therefore, we present a novel Convolutional Neural Network (CNN) for yield prediction called Hyper3DNetReg. Our Hyper3DNetReg network takes a data cube as the input, which can be interpreted as a multi-channel image corresponding to a small neighborhood of points in the field. Each channel of this input data cube represents a different feature of that neighborhood (e.g., nitrogen rate applied). Given a two-dimensional input with multiple channels, Hyper3DNetReg outputs a two-dimensional raster, where each pixel output estimates the yield value of its corresponding input pixel. Significantly, our models are field-specific, which means they are trained on data of a given field and used to predict future yield maps using data of the same crop in the same field from a year different than those that were used for training. Given this, we hypothesized that our Hyper3DNetReg network with a two-dimensional output would lead us to generate more accurate predicted yield maps compared to several compared methods that utilize a single output. Accuracy was assumed to be based on the reproduction of yield values from combine yield monitors.

This paper is an extension of a non-refereed paper originally presented at the SPIE Future Sensing Technologies conference [21]. Extensions include an extensive set of experiments that allows for a broader analysis of the behavior of the proposed yield prediction method. In particular, we performed a leave-one-year-out cross-validation design in order to study how our method compares to other yield prediction methods when given good or poor data representativeness in the training set. For example, we analyzed how different models behave when given training information from only dry years and tried to generalize for wetter years. We also considered the case where the models are trained using information combined from both dry and wet years. Furthermore, we included two additional yield prediction methods for comparison, namely random forest and Bayesian multiple linear regression.

This paper is structured as follows: Section 2 provides a review of previous work done on modeling yield response to crop management. In Section 3.1, we describe the winter wheat datasets used in this work and the techniques used for their pre-processing. Section 3.2, Section 3.3 and Section 3.4, provide details about our yield prediction models, the Hyper3DNetReg network architecture, and the proposed yield map generation method. Experimental results are reported in Section 4 and their implications are discussed in Section 5. Finally, Section 6 offers concluding remarks.

## 2. Related Work

Crop yield prediction is one of the most studied tasks of PA. Classic approaches involve the use of parametric functions that attempt to model the crop yield response to input variables, such as nitrogen. Response functions can be modeled as polynomial functions, for instance, linear, quadratic, or exponential [22,23]. They can also be based on basic agronomic principles, as is the case with the Liebig response functions [24]. Other works have modified typical response functions to allow certain parameters to be stochastic, which led to better results than their deterministic counterparts [25]. What is more, some studies consider the spatial correlation among neighboring crop yield responses within the field. Hence, they demonstrate that N response differs by landscape position and suggest that site-specific application may increase net returns [13,26].

From the machine learning standpoint, crop yield response functions are learned based on observed data. By doing so, we avoid assuming generic parametric response functions that may fail at capturing different local behaviors within the field. Previous works are commonly based on the use of linear regression [16,27] and non-linear regression models [28]. They may rely on remotely sensed covariates, such as vegetation indexes calculated from satellite imagery (e.g., Normalized Difference Vegetation Index (NDVI) and Normalized Difference Water Index (NDWI)) [27,28]. Remotely sensed data can also be combined with on-ground data. Usually, complex multivariate regression problems require more advanced machine learning techniques, such as k-nearest neighbors [29] regression trees [30], support vector machines [31], and random forests [32].

In recent years, many artificial neural network-based approaches have been proposed for crop yield prediction. For instance, Peerlinck et al. [20] designed deep neural networks for yield and protein prediction based on feedforward networks (FNN) and stacked autoencoders (SAE) using data consisting of factors such as previous years’ nitrogen application rate, field slope, NDVI, and precipitation. As part of this work, they also explored spatial sampling in order to account for the mutual dependency between neighboring points in the field. Their spatial sampling technique consisted of using the eight nearest neighbors of each point in the field. Their non-spatial results showed that FNNs and SAEs outperformed shallow neural networks and simple linear and non-linear regression models. The spatial results showed that FNNs and SAEs had even better performance than the non-spatial versions. Subsequently, Peerlinck et al. [18] also proposed to use an Approximation AdaBoost algorithm with feedforward networks as weak learners. These networks also used the spatial sampling from the earlier work. This method approximates the loss function by using a threshold approach that discards small errors during the weight update of the weak learners. Experimental results showed that this approach gave better results than using simple FNNs with a single hidden layer.

A CNN is a specialized type of artificial neural network designed for processing data with a grid-like topology. Their convolutional filters provide a way to take explicit advantage of the spatial information of small regions of the field while avoiding the use of an excessive amount of parameters. This is because CNNs allow for parameter sharing, which refers to the fact that the same set of values of a convolutional filter is applied at every position of the input instead of learning a separate set of parameters for every location [33]. In addition, recurrent neural networks, such as the Long-Short Term Memory (LSTM) networks, allow for incorporating temporal information into the models. Therefore, You et al. [34] presented a crop yield prediction method based on the combination of LSTMs and CNNs. To do this, they proposed to model the spatio-temporal structure of the data using Gaussian processes. The limitation of this approach is that it requires manually finding the optimal time frame that will be used to extract the training data for each field. In addition, the method cannot be applied in regions with scarce satellite data due to recurrent occlusions (e.g., clouds and smoke) and limited satellite coverage. In order to overcome these limitations, Russello [15] proposed a 3-D CNN architecture that leverages spatio-temporal features. The input of this network consists of the concatenation of multiple satellite image patches collected across a determined amount of time. In other words, the third dimension of this “data cube” accounts for the temporal information of the data. Russello demonstrated that the 3-D CNN network outperformed the one proposed by You et al. [34] as well as other classic machine learning techniques.

Conversely, Barbosa et al. [19] presented a CNN-based approach that does not use temporal information; that is, the explanatory variables correspond to a unique period of time. Specifically, it uses data collected right after soil tillage. As a consequence, the proposed CNN architecture is two-dimensional (2-D), unlike the aforementioned approaches. Their input consists of a set of five raster covariates: nitrogen rate, seed rate, elevation map, and soil’s shallow electroconductivity. They suggested using small networks to reduce the computational complexity that is usually associated with training deep learning models. Thus, they proposed a multi-stream network called CNN-Late Fusion (CNN-LF), where each covariate is connected to an independent CNN (i.e., a stream). Then, the outputs from the independent streams are fused into a single predicted yield value using fully connected layers. It is important to point out that all the previously mentioned works output a single predicted yield value that corresponds to one specific cell of the field. Even in the case of NNs that use spatially-sampled data or CNNs with spatial neighborhoods as inputs, they only predict the yield value of the central cell of the input neighborhood.

Finally, the use of Synthetic Aperture Radar (SAR) images for predicting yield was explored in this work. SARs are active sensors and, as such, the information they capture is insensitive to atmospheric conditions given that the microwaves they emit are able to penetrate through clouds. This represents an important advantage over optical satellite images, whose availability can be affected by the presence of clouds and whose reflectance values can be distorted due to smog or smoke. Backscattering coefficients of SAR images are directly related to the dielectric properties of the soil surface being observed [35]. Dielectric properties are mainly dependent on the moisture content of the soil, thus, SAR images can be used for soil moisture retrieval [36,37]. Throughout this work, we used SAR images captured by the Sentinel-1 mission, given their easy accessibility for farmers. Previous works built prediction models to estimate the production of winter wheat, rapeseed, and rice crops using a series of Sentinel-1 images that describe the evolution of the crops during the entire growing season [38,39]. Furthermore, Zhuo et al. [40] derived time series of soil moisture rasters from Sentinel-1 and Sentinel-2 images using the water cloud model [41]. This information was then incorporated into the World Food Studies (WOFOST) crop model [42] to improve the yield estimation accuracy of winter wheat.

## 3. Materials and Methods

### 3.1. Datasets

**Figure 1:**
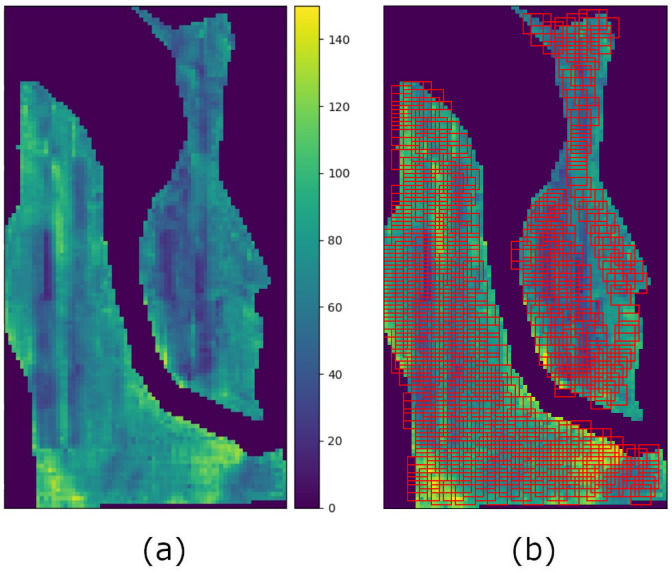
Image rasters corresponding to the G1 field (2018). (**a**) Yield raster. (**b**) Automatic extraction of 5×5 pixel patches.

For our experiments, we collected data from four fields of winter wheat. The fields are located on two different farms in Montana with different climates and soils. Yield maps were acquired during the harvest season, which corresponded to the month of August for all years and fields of this study. Site-specific yield values were measured in bushels per acre (bu/ac) by a yield monitor mounted on a combine harvester. Every three seconds while traveling through the field, the yield monitor measured the volume rate of harvested material and integrated this flow rate to generate an estimate of the total amount of harvested material during that interval. The generated yield data values were then georeferenced with a location provided by an onboard GPS. Finally, grid-like yield maps with equally spaced points were aggregated at a scale of 10 m. Considering that multiple points could be found within a 10× 10 m cell, we used the median to represent the yield value of that cell. Note that the median was preferred over the mean given that it is more robust to outliers. Figure 1a. shows an example of an aggregated yield map for one of our fields. Each pixel represents a cell of the field and, as such, the yield map can be seen as an image raster.

While the yield value represents the response variable in our regression problem, the explanatory variables corresponded to a combination of remotely sensed data and on-ground data. Among the remotely sensed data, we considered SAR satellite images acquired by Sentinel-1. Sentinel-1 images contain two bands acquired using Vertical Transmit-Vertical Receive Polarization (VV) and Vertical Transmit-Horizontal Receive Polarization (VH). These images were obtained at the Ground Range Detected (GRD) level, which includes three pre-processing steps: speckle noise reduction, thermal noise removal, radiometric calibration, and ortho-rectification [43]. The resulting images have a spatial resolution of 10 m.

Besides the Sentinel-1 images, the collected dataset consists of a set of six raster features: nitrogen rate applied (lbs/ac), annual precipitation (mm), slope (degrees), elevation (m), topographic position index (TPI), and terrain aspect (radians). The nitrogen rate applied is gathered from the farmer’s application equipment and aggregated to the 10 m in the same manner as to yield responses, while precipitation is gathered at a 1 km scale from NASA’s Daymet V3 dataset and georeferenced to pixels via the intersection of 10 m points within the ∼1 km pixels. Note that we measure the amount of precipitation during the current water year; that is, from November 1st to March 30th. Topographic variables are gathered at a 10 m scale from the USGS Digital Elevation Model. Thus, our resulting input can be viewed as an image data cube with eight channels in total where each pixel has a resolution of 10 × 10 m (i.e., the same resolution as our yield maps). It is of particular importance to note that the nitrogen fertilizer was top-dress applied in March and the Sentinel-1 images were selected from the same month. Thus, we used data acquired in March to predict crop combine yield monitor values in August of the same year.

Given that each field is represented with one large raster image, we must divide it into small 2-D patches that will be processed by our CNN regression model. To do this, we extract square patches using a 5×5 pixel window with a maximum overlap of 19 pixels (75% of the pixels of the window) so that we collect a sufficient number of training samples from one single field Figure 1b. Table 1 shows the total number of extracted samples and the years of observation for each field used in our experiments. Here, fields starting with the same letter (i.e., G1, G2, and G3) belong to the same farm (The names of the farms and fields were hidden in order to protect farmers’ privacy). Furthermore, we evaluate the performance of our models in a similar way to leave-one-out cross-validation, which we refer to as “leave-one-year-out” cross-validation. More specifically, given a field, we repeat the training process as many times as observed years available using each year as an independent test set while using the remaining years to construct our training and validation sets (we use 90% of the data for training and 10% for validation). Table 2 shows how the data is split for each field.

### 3.2. Yield Prediction Model

Let *X* and *Y* denote the input and target output of our prediction model, respectively. *X* is an image data cube of W×W pixels with *n* channels. Note that each channel corresponds to a different covariate. As explained in Section 3.1, we set W=5 and n=8 for the datasets used in this work. Output *Y* is a two-dimensional image of N×N pixels (N≤W) that corresponds to the ground-truth yield; that is, the yield observed during the harvest season. Here, the value of the (i,j)–th pixel of *Y* is the observed yield, corresponding to the $\left(i+\frac{W-N}{2},j+\frac{W-N}{2}\right)$–th pixel of *X*.

We constructed regression models that were trained to capture the association between the target *Y* and the input *X* using our Hyper3DNetReg CNN architecture. Let the function computed by Hyper3DNetReg and their corresponding weights be denoted by f(·) and θ, respectively. Thus, the predicted yield $\hat{Y}$ given input *X* is calculated as:

$$\hat{Y}=f\left(X\right).$$

**Figure 2:**
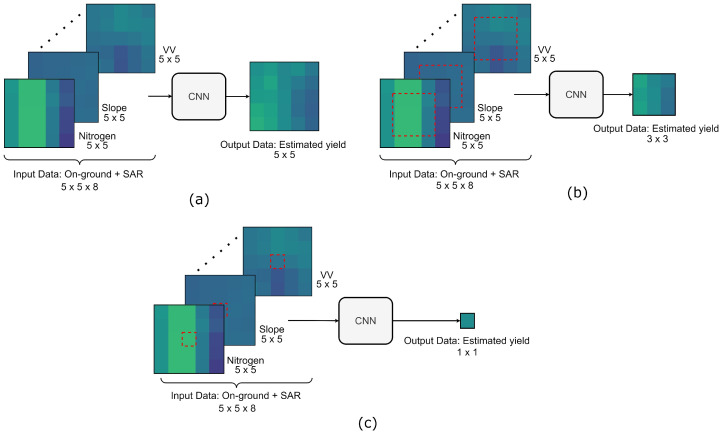
Proposed yield prediction model using different output window sizes: (**a**) 5×5, (**b**) 3×3, and (**c**) 1×1.

We implement models using three output window sizes (i.e., N=5,3,1) to evaluate the impact of *N* on the effectiveness of our method. Figure 2 depicts our proposed yield prediction models. The output windows shown in Figure 2b,c represent the predicted yield of the area enclosed by the dotted squares within the input images.

### 3.3. Hyper3DNetReg Architecture

In this section, we present our proposed CNN architecture for crop yield prediction called Hyper3DNetReg. It is a 3D–2D CNN architecture (i.e., it uses three-dimensional and two-dimensional convolutional layers) that is based on a network we previously proposed called Hyper3DNe [44]. The Hyper3DNet network receives as inputs hyperspectral data cubes and was designed to solve hyperspectral classification tasks. Even though the yield prediction problem differs from hyperspectral classification, we propose to adapt the Hyper3DNet architecture for 2-D regression. Note that, in this work, we use two-dimensional inputs with multiple channels that can be interpreted as data cubes (see Figure 2), as in the case of hyperspectral images. Thus, we can take advantage of the 3-D convolutional filters used by the Hyper3DNet network in order to capture not only the spatial information of the input neighborhoods but also the interaction between input covariates.

We shall point out the main aspects that prevent us from using the original Hyper3DNet architecture directly. On the one hand, the Hyper3DNet is a classification network while the crop yield prediction is a regression problem. The output of the Hyper3DNet network is a one-dimensional vector containing real-valued numbers bounded between 0 and 1 corresponding to the scores of each of the possible classes (the set of classes is finite). Conversely, a regression problem is not associated with pre-determined class labels; instead, its unique real-valued output represents an estimate of the target output so that its values are not necessarily bounded as in the case of the class labels. In addition, the output of our regression problem consists of a two-dimensional patch with dimensions N×N (where N= 5 or 3) or a single real value (N=1), as explained in Section 3.2. Therefore, the Hyper3DNet architecture needs to be modified to fit our needs.

Table 3 shows the architecture of the Hyper3DNetReg network. Note that the general input shape of our network is (5,5,n,1), which indicates that our input consists of a single data cube with a width and height of five pixels, and *n* input channels (i.e., the number of covariates). The Hyper3DNetReg network can be divided into two main modules: 3-D feature extractor and 2-D encoder. The former consists of four densely connected blocks [45] with 3-D convolutional layers (denoted as “Conv3D”), whose layers are connected directly using skip connections to ensure maximum information flow throughout the network. The skip connections allow for concatenating the outputs (using “CONCAT” layers) of the two preceding layers along the fourth dimension. Each convolutional layer is followed by a rectified linear unit activation layer, denoted as “ReLU” (where ReLU(x)=max(0,x)), and a batch normalization layer, denoted as “BN”.

**Figure 3:**
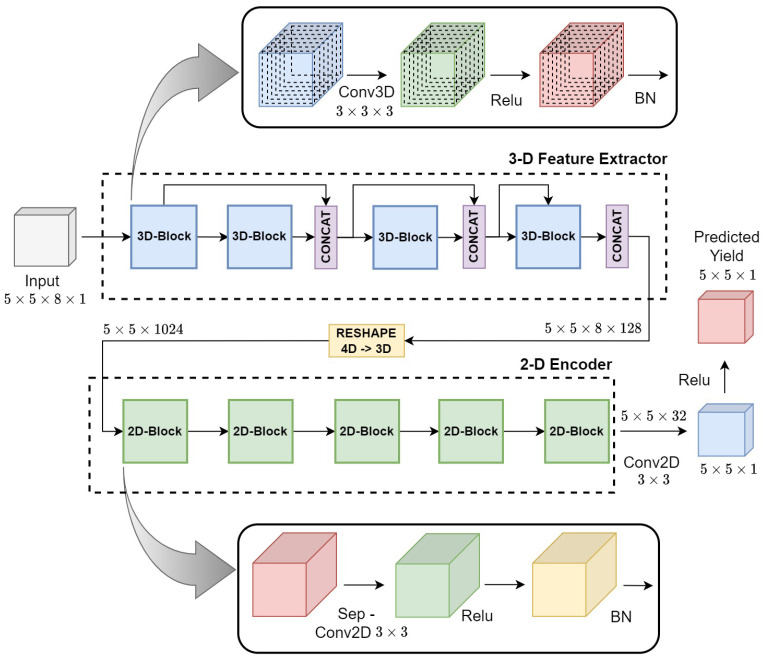
Hyper3DNetReg architecture with a 5×5×8×1 input and a 5×5×1 output.

The second module of the network, the 2-D encoder, consists of five 2-D separable convolution layers [46] (denoted as “SepConv2D”), which allow performing 2-D convolution operations while avoiding using an excessive number of trainable parameters [47]. Notice that the output of the 3-D feature extractor needs to be reshaped into a 3-D tensor before it can be processed by the SepConv2D layer. We included “Dropout” units between layers of the 2-D encoder. Only during training, these units zero out some elements of the preceding tensor with a probability of 0.5 to help prevent overfitting [48]. Finally, if the output is a two-dimensional patch (N= 5 or 3), the last layer of the network is a simple 2-D convolutional layer (denoted as “Conv2D”), which is used to reduce the number of channels to 1 and to adjust the output window size if needed. Otherwise, if the output is a single predicted value (N=1), the last layer is a fully connected layer, denoted as “FC”. Figure 3 illustrates our network architecture using a 2-D output.

It is worth mentioning that the 2-D encoder of Hyper3DNetReg differs from that of Hyper3DNet in that their SepConv2D layers do not shrink the spatial dimensions of the tensors throughout the network. We achieved this by using a stride and padding of (1,1), considering that we use 3×3-pixel and 3×3×3-pixel kernels for all 2-D and 3-D convolutional layers of the network, respectively. The reason to do this is that, in the case that we desire to obtain an output window size of N=5 (i.e., the output has the same spatial dimensions as the input), we must assure that the spatial dimensions of the tensors generated by the inner blocks of the network are not affected by any downsampling and always remain the same. Another difference between Hyper3DNetReg and Hyper3DNet is that, if the output is 2-D, Hyper3DNetReg uses a Conv2D layer instead of the FC layer used by Hyper3DNet. This helps to reduce the number of trainable parameters. For example, consider that the output window size is N=5, then the last Conv2D layer would require 289 parameters. Conversely, if we instead decided to use an FC layer with 25 outputs (a vector of 25 elements can be considered the vectorized representation of a 5×5 matrix), this layer would require 20,025 parameters. Note that in the case that N=1, we can use an FC layer as the final layer as it involves the use of only nine additional parameters.

### 3.4. Predicted Yield Map Generation

As explained in Section 3.2, the input of our models has a spatial dimension of 5×5 pixels, which represents an area of 50×50 m in the field. This allows us to obtain yield predictions for local neighborhoods; nevertheless, our objective is to generate predicted yield maps for the entire field. In order to do so, we examine the surrounding 5×5-pixel neighborhood of each cell in the field and process it through our Hyper3DNetReg network. This can be interpreted as moving a sliding window of 5×5 pixels throughout the entire field to obtain yield estimations at each cell. Note, however, that if the network output is designed to be two-dimensional (N= 3 or 5), the predicted yield windows of consecutive points will overlap.

**Figure 4:**
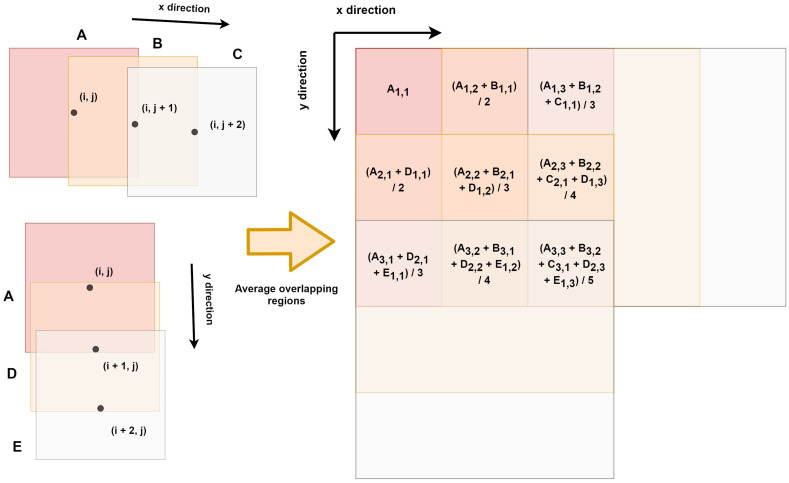
Yield map generation using overlapping patches.

Let us consider a cell of the field with coordinates (i,j), as shown in Figure 4. In this example, the cell (i,j) has only two neighbors on the right side, whose coordinates are (i,j+1) and (i,j+2), and two neighbors below, whose coordinates are (i+1,j), and (i+2,j). Thus, we generated the predicted yield windows A, B, C, D, and E for cells (i,j), (i,j+1), (i,j+2), (i+1,j), and (i+2,j), respectively. Considering that window A overlaps with the other windows, the aggregated predicted yield map is obtained by averaging the predicted yield values of the overlapping regions. It is important to notice that, in practice, when we analyze a cell of the field, we use all available neighbors whose yield prediction windows overlap with that of our cell of interest. For example, if N=5, a pixel of a yield estimation window may overlap with up to 25 windows. Therefore, we obtain several predicted yield values for each cell of the field using slightly different neighborhoods. We argue that, by doing so, we mitigate noisy results and generate smoothed predicted yield maps.

## 4. Results

The objective of this paper is to evaluate the performance of our Hyper3DNetReg network in comparison to other machine learning-based crop yield prediction techniques. As such, our experiments tested our methodology against seven other methods in each field: Approximate AdaBoost (AdaBoost.App) [18], a stacked autoencoder (SAE) [20], a three-dimensional CNN (3D-CNN) [15], CNN-Late Fusion (CNN-LF) [19], random forest (RF), Bayesian multiple linear regression (BMLR), and multiple linear regression (MLR). We acknowledge that different fields (especially those corresponding to different farms) behave differently. This is supported by the fact that it has been observed that yield response is highly dependent on factors such as weather and soil composition, which vary considerably from field to field [13], all experiments in this paper are field-specific; that is, a model is trained on data of a field and tested on data of the same field but from a different year than those used for training, according to the data distribution explained in Table 2.

We implemented the compared methods and used the hyperparameters suggested by their authors in their corresponding code repositories. Nevertheless, the characteristics of our data do not necessarily match those of the data used by the compared methods originally. Thus, we adapted the methods as needed and tuned some of their hyperparameters to obtain the best possible performance and achieve a fair comparison. We briefly describe their most important aspects. For example, the set of weak learners used for AdaBoost.App consists of ten FNNs with three hidden layers each. These layers consisted of 100, 96, and 32 hidden nodes. Similarly, we used a three-hidden-layer FNN for SAE. In this case, a single network is trained and thus its complexity should be greater than that of the weak learners used by AdaBoost.App. As such, their layers consisted of 500, 250, and 125 nodes, respectively. For RF, we used 500 trees, where each could be expanded up to a maximum depth of 10.

Furthermore, the 3D-CNN proposed by Russello [15] was designed to leverage spatio-temporal features, as explained in Section 2. Its original architecture has an input shape of 64×64×10×24 pixels. This can be read as an input that consists of 24 data cubes (captured over 24 different days across the growing season) with a height and width of 64 pixels, and 10 input covariate factors. This differs from our approach in that we collect a single data cube right before the fertilizer application; besides, we consider inputs with a height and width of five pixels, and eight input covariate factors. Therefore, the 3D-CNN was adapted to process inputs with dimensions of 5×5×8×1 pixels and output single predicted yield values. On the other hand, the CNN-LF method also collects data from a specific moment of the growing season. However, its first layer is a 2-D convolutional layer that takes an input of 21×21×5 pixels, which in this case is read as a set of five covariate rasters with a height and width of 21 pixels. Thus, we reshaped the samples of our datasets from 4 to D to 3-D tensors and adapted the CNN-LF architecture to accept our input dimensions. The rest of the methods, namely AdaBoost.App, SAE, RF, BMLR, and MLR take as inputs one-dimensional vectors of eight elements. For that reason, we vectorized our datasets so that each sample consists of a vector of eight elements. It is important to mention that, unlike our Hyper3DNetReg architecture, all of these methods produce a single yield value regardless of the input dimensions.

All methods were trained using the same configuration for the sake of consistency and fairness. While this strategy does not guarantee the best possible results, it allows us to compare the behavior of different prediction methods under the same conditions. That is, they were trained using the same objective function and data distribution (i.e., the same training and validation sets). Regression models were trained to generate accurate estimates $\hat{Y}$ with respect to *Y*; thus, their training objective was to minimize the mean squared error (MSE) of the estimation. In addition, neural network-based methods (Hyper3DNetReg, AdaBoost.App, SAE, 3D-CNN, and CNN-LF) used the same optimizer and mini-batch size. The selected optimizer was Adadelta [49], which is an adaptive gradient descent method that requires no manual tuning of a learning rate. The mini-batch size for all the fields was set to 96.

Each covariate in our dataset represents a different type of information and, as such, their range of values is not necessarily the same. For example, the terrain aspect is measured in radians so that its values are bounded between 0 and 2π, while the nitrogen fertilizer rate is measured in pounds per acre and its values are bounded between 0 and 150. Therefore, the range of all covariates is normalized using min-max normalization. Note that normalization is performed based on the information provided in the training sets. During validation and testing, we apply the scaling that was calculated previously.

We compared all crop yield prediction methods based on performance metrics calculated on their generated yield prediction maps. As such, given a field and a split selected from Table 2, each of the eight methods used the information from the training and validation sets to fit a model. Then, the predicted yield map was generated on the corresponding test set using the fitted model according to the process described in Section 3.4. We considered four performance metrics to compare the generated yield prediction map (*P*) and the observed yield map (*M*), also known as combine yield monitor map. The metrics are: Root mean square error (RMSE), root median square error (RMedSE), Pearson correlation coefficient (*r*), and structural similarity (SSIM). RMSE and RMedSE are calculated as follows:

$$RMSE=\sqrt{\frac{\sum_{(i,j)\in F}{\left(M\left(i,j\right)-P\left(i,j\right)\right)}^{2}}{\left|F\right|}},$$

$$RMedSE=\sqrt{\underset{(i,j)\in F}{median}{\left(M\left(i,j\right)-P\left(i,j\right)\right)}^{2}}.$$

The Pearson correlation coefficient is defined as the normalized covariance between two variables; hence, its values are bounded between −1 and 1. When used as a performance metric for yield regression, it conveys the strength of the linear correlation between the observed and predicted yield values:

$$r=\frac{\sum_{(i,j)\in F}\left(M\left(i,j\right)-\bar{M}\right)\left(P\left(i,j\right)-\bar{P}\right)}{\sqrt{\sum_{(i,j)\in F}{\left(M\left(i,j\right)-\bar{M}\right)}^{2}}\sqrt{\sum_{(i,j)\in F}{\left(P\left(i,j\right)-\bar{P}\right)}^{2}}},$$

where $\bar{M}=\frac{1}{\left|F\right|}\sum_{(i,j)\in F}M\left(i,j\right)$ and $\bar{P}=\frac{1}{\left|F\right|}\sum_{(i,j)\in F}P\left(i,j\right)$ represent the mean values of the observed and predicted yield maps, respectively. In the ideal case that the prediction error is minimum (|M−P|≈0), we would expect a strong linear correlation between *M* and *P* and thus the correlation coefficient would be r≈1.

**Figure 5:**
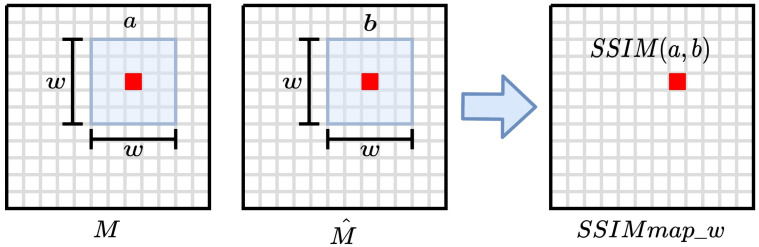
Demonstration of the SSIM map generation between images *M* and *P*. SSIM(a,b) represents the SSIM value between the local windows *a* and *b*.

Furthermore, note that a yield map is not an image per se; however, we can treat it as such given its grid-like structure. Hence, the yield value of a cell can be interpreted as a pixel intensity value. The task of comparing the observed map *M* and the predicted map *P* can now be approached as an image similarity task. In this context, SSIM is a common metric for image quality assessment [50]. It is used to compare a noisy image with a reference image (i.e., without noise) taking into consideration three aspects: luminance, contrast, and structure. First, we define the SSIM index as a local metric that is used to compare the similarity of two small windows of w×w pixels from *M* and *P*, as illustrated in Figure 5. The SSIM index is a single number that can take values between −1, which indicates perfect anti-correlation, and 1, which indicates that both windows are identical. Moreover, a w×w sliding window can be displaced throughout all the pixels of the image maps to create an SSIM quality map.

The size of the sliding window *w* determines the level of detail considered by the SSIM index. That is, small windows focus on granular details while large windows capture bigger structures. Since we are interested in studying both cases, we produced two SSIM maps for comparing *M* and *P*, SSIMmap_3 and SSIMmap_11, using w=3 and w=11, respectively. Notice that SSIMmap_3 and SSIMmap_11 have the same dimensions as the yield maps and thus are not adequate to be used as a performance metric Therefore, we defined the metrics SSIM3 and SSIM11 that summarize the information provided by SSIMmap_3 and SSIMmap_11:

$$SSIM3=\frac{1}{\left|F\right|}\sum_{(i,j)\in F}SSIMmap\_3,$$

$$SSIM11=\frac{1}{\left|F\right|}\sum_{(i,j)\in F}SSIMmap\_11.$$

**Figure 6:**
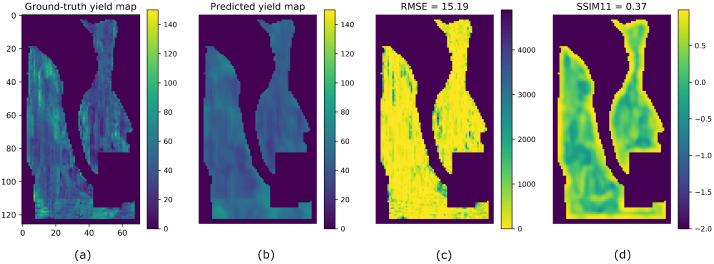
Yield prediction example of the G1 field for the year 2020. (**a**) Ground-truth yield map. (**b**) Predicted yield map using our Hyper3DNetReg with N=5. (**c**) Square error map. (**d**) SSIMmap_11.

Let us consider the split A of the G1 field as an example. According to Table 2, the training and validation sets correspond to data collected from 2016 and 2018, while the test set corresponds to data collected from 2020. The test set is used to create the observed yield map *M*, which is depicted in Figure 6a. We trained our Hyper3DNetReg network using an output window size of N=5. The resulting model was then evaluated on the test set and a predicted yield map was generated using the methodology explained in Section 3.4 (see Figure 6b). In addition, we constructed the square error map shown in Figure 6c, defined as ${\left(M-P\right)}^{2}$, in order to visualize the spatial distribution of the differences between the observed and predicted values. We also generated the SSIMmap_11 map (Figure 6d) between *M* and *P* to identify the regions with high and low structural similarity. Figure 6d shows that the SSIM index is greater near the border of the field. This can be explained by the fact that the 11×11 windows generated near the border include zero-valued pixels (i.e., pixels located outside the borders of the field). As a consequence, the local windows extracted from both *M* and *P* contain zero-valued pixels at the same locations, which makes them structurally similar and causes the resulting SSIM index to be high.

**Figure 7:**
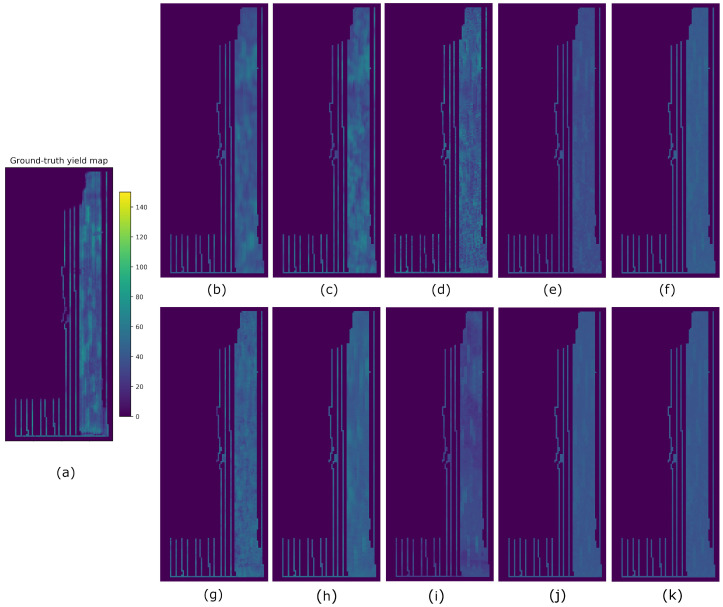
Yield prediction comparison on the F1 field using split A. (**a**) Ground-truth. (**b**) Hyper3DNetReg (N=5). (**c**) Hyper3DNetReg (N=3). (**d**) Hyper3DNetReg (N=1). (**e**) AdaBoost.App. (**f**) SAE. (**g**) 3D-CNN. (**h**) CNN-LF. (**i**) RF. (**j**) BMLR. (**k**) MLR.

**Figure 8:**
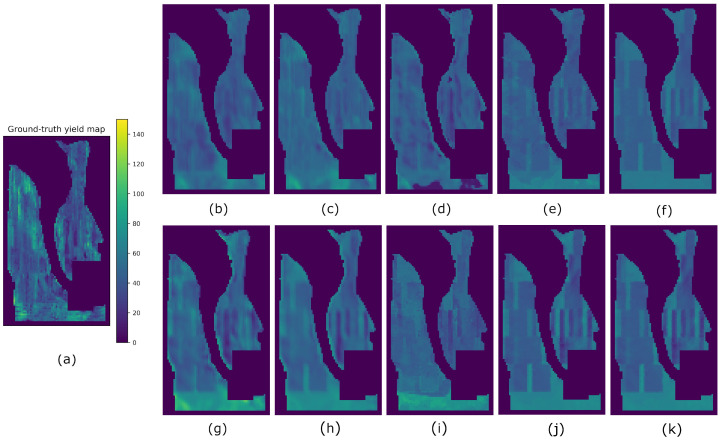
Yield prediction comparison on the G1 field using split A. (**a**) Ground-truth. (**b**) Hyper3DNetReg (N=5). (**c**) Hyper3DNetReg (N=3). (**d**) Hyper3DNetReg (N=1). (**e**) AdaBoost.App. (**f**) SAE. (**g**) 3D-CNN. (**h**) CNN-LF. (**i**) RF. (**j**) BMLR. (**k**) MLR.

**Figure 9:**
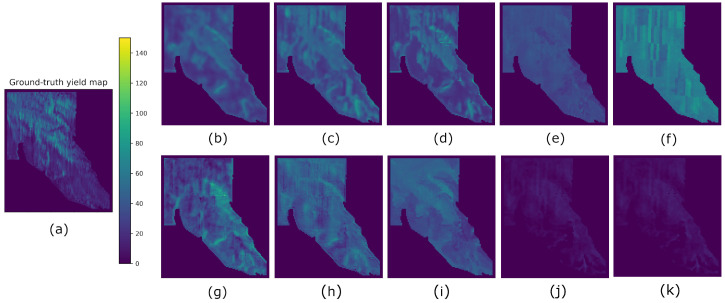
Yield prediction comparison on the G2 field using split A. (**a**) Ground-truth. (**b**) Hyper3DNetReg (N=5). (**c**) Hyper3DNetReg (N=3). (**d**) Hyper3DNetReg (N=1). (**e**) AdaBoost.App. (**f**) SAE. (**g**) 3D-CNN. (**h**) CNN-LF. (**i**) RF. (**j**) BMLR. (**k**) MLR.

**Figure 10:**
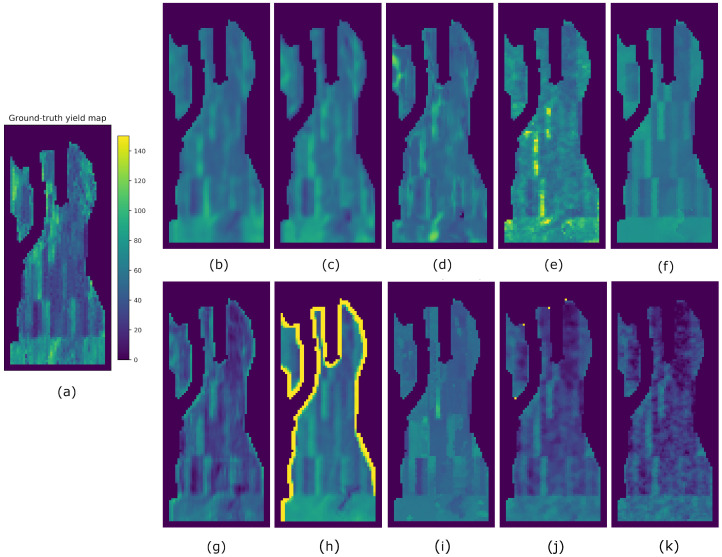
Yield prediction comparison on the G3 field using split A. (**a**) Ground-truth. (**b**) Hyper3DNetReg (N=5). (**c**) Hyper3DNetReg (N=3). (**d**) Hyper3DNetReg (N=1). (**e**) AdaBoost.App. (**f**) SAE. (**g**) 3D-CNN. (**h**) CNN-LF. (**i**) RF. (**j**) BMLR. (**k**) MLR.

Results from all methods and fields using splits A, B, and C are reported in Table 4, Table 5 and Table 6, respectively. The best-performing metrics obtained for each field are highlighted in bold. For easier visualization and comparison of the results, we multiplied the averaged SSIM indices by 100 (denoted as $SSIM3^{*}$ and $SSIM11^{*}$). In order to compare the predicted yield maps obtained by the eight compared methods visually, we took the results obtained from split A. Hence, the yield maps generated for fields F1, G2, G1, and G3 are shown in Figure 7, Figure 8, Figure 9 and Figure 10, respectively.

## 5. Discussion

From comparing Table 4, Table 5 and Table 6, we noticed that the results from Table 5 (split B) showed the poorest performance for all the fields. This can be explained by looking at Table 7, which shows the annual average precipitation per field. Note that the second year of the study corresponds to the wettest year for all fields. Hence, split B used two dry years for training (one, for the case of G3) and a wet year for testing. The F1 field suffered from a similar problem when using split C. In this case, two wet years are used for training and a dry year for testing. Due to poor data representativeness in the training set, the trained models were not able to generalize correctly considering that the amount of precipitation is a crucial factor for crop development and growth. Models trained on datasets with insufficient data representativeness tend to overestimate or underestimate the predicted yield.

Thus, these cases should be analyzed carefully depending on the context. For example, in Table 6, MLR performed better than the other methods in the F1 field. The *p*-value obtained by the MLR model for the precipitation covariate is $4\times 10^{-75}$, which means it did not consider that there is correlation between precipitation and yield. On the other hand, the other methods (except for CNN-LF) did not assume that the covariates were independent and thus considered the precipitation information during training. During testing, these models used a precipitation value of 86 mm, which was lower than what they used for training (101 and 130 mm, according to Table 7). Therefore, their predictions were biased toward high yield values, which caused higher errors in comparison to MLR.

**Figure 11:**
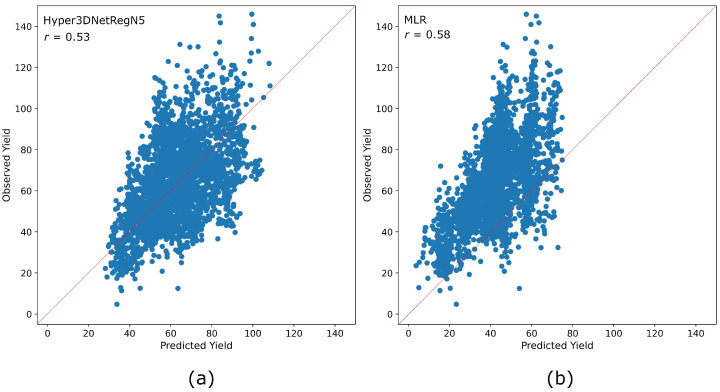
Predicted yield vs. observed yield plots obtained for the G3 field (Split B). (**a**) Results obtained by Hyper3DNetReg (N=5). (**b**) Results obtained by MLR.

The correlation coefficient *r*, which is directly related to the coefficient of determination $R^{2}$, is commonly used in crop yield prediction tasks as a performance metric [10]. Nevertheless, we argue that it does not always entail a good measure of the goodness-of-fit of a model to data. The reason is that *r* is scale and offset invariant. In addition, it is highly susceptible to the presence of outliers [51]. Hyper3DNetReg always achieved one of the highest *r* correlation coefficient values from Table 4, Table 5 and Table 6. However, there are cases where methods with considerably higher RMSE values achieved better *r* values than Hyper3DNetReg. For instance, note from Table 5 that MLR obtained a higher *r* value than Hyper3DNetReg with N=5 for field G3 despite of obtaining a higher RMSE value. In Figure 11, we show the predicted yield vs. observed yield plots obtained by Hyper3DNetReg and MLR. From this, we see that even though the results obtained by MLR lean to the left side (i.e., MLR underestimates the yield response), both cases show a linear relationship, and, what is more, MLR produces a slightly higher correlation coefficient. Because of these reasons, we consider the correlation coefficient *r* as an unreliable performance metric for a nonlinear regression task such as ours.

The case where the Hyper3DNetReg network uses an output window size of five pixels (N=5) is denoted as Hyper3DNetReg-N5 for conciseness. Similarly, the configurations of Hyper3DNetReg with output sizes of three pixels and one pixel are denoted as Hyper3DNetReg-N3 and Hyper3DNetReg-N1, respectively. From the cases with reasonable data representativeness (Table 4 and Table 6), we conclude that our Hyper3DNetReg-N5 achieved the best results in the fields studied. Take the results obtained for field F1 from Table 4 as an example. Here, we notice that Hyper3DNetReg-N5, SAE, CNN-LF, and MLR achieved similar RMSE values (10.88, 10.43, 10.73, and 10.98, respectively). Nevertheless, the analysis of the results is not limited to finding the method with the lowest RMSE value. We also take into account the SSIM metrics as they provide information about the structural similarity between the predicted yield map and the observed yield map. As such, a high overall structural similarity value allows us to verify that regions of high and low predicted yield correspond to actual regions of high and low observed yield. By considering the RMSE metric together with the SSIM3 and SSIM11 metrics, we concluded that Hyper3DNetReg-N5 produced one of the lowest RMSE and also the highest SSIM3 and SSIM11 values. As a consequence, we conclude the Hyper3DNetReg-N5 is the best prediction method for this field.

**Figure 12:**
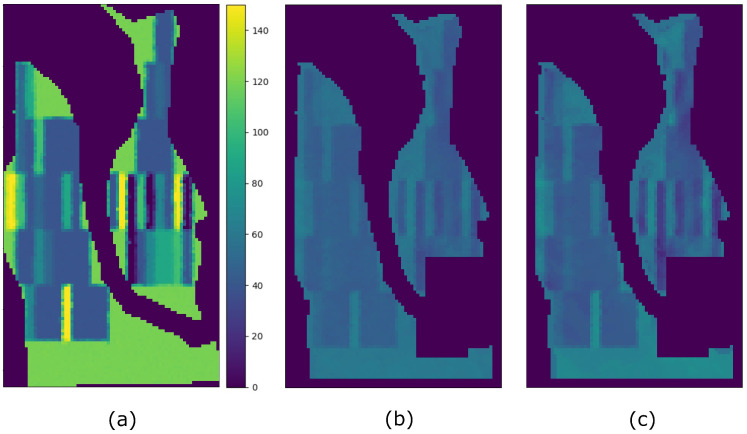
Comparison of the nitrogen rate map and the predicted yield map on the G1 field. (**a**) Nitrogen rate map. (**b**) SAE. (**c**) MLR.

It is worth mentioning that other methods may produce higher SSIM values than Hyper3DNetReg-N5, as is the case of SAE when analyzing the G1 field and using split A (Table 4). From Figure 8f, we observe that the yield map generated by SAE presented some high-contrast regions that are structurally similar to the observed yield map, which increased the value of the SSIM metrics. As observed, this may happen even though the predicted yield values do not necessarily coincide with the observed yield values, which entails a high RMSE. The map generated by Hyper3DNetReg-N5 (Figure 8a) is smoothed or blurred because of the process described in Section 3.4 that averages overlapping regions. As a consequence, its corresponding SSIM metrics are lower despite also detecting the same high-contrast regions. Furthermore, the reason why SAE (Figure 8e), AdaBoost.App (Figure 8f), and MLR (Figure 8k) produced sharp-edge shapes is that, after training, they considered the nitrogen fertilizer rat as the most relevant covariate. Therefore, the corresponding predicted yield maps were highly influenced by the nitrogen rate maps, showing high structural similarity, as depicted in Figure 12. The fields in this study had spatially distributed nitrogen rates to develop the functional relationships between nitrogen fertilizer and crop yield and thus was logically a factor that should be included in further development of our method. Future work will focus on measuring the relevance each variable has when predicting yield values.

The G3 field was a special case because it had fewer training samples than the rest of the fields and only one year of training data. In addition, the training set of split A only included patch samples from the inner regions of the field and not from the regions next to the boundary because they had missing nitrogen and yield information. Thus, the limited amount of training data and its lack of diversity caused the models to be prone to overfitting. This can be seen with CNN-LF (see Figure 10h) where the overfitting arises due to the fact CNN-LF is a multi-stream network that analyzes each covariate separately and fuses the outputs from each stream using a single neuron. In this case, the final neuron assigned a large negative weight to the stream that corresponded to the precipitation covariate. Because of the negative correlation estimated by CNN-LF between yield and precipitation, it predicted higher yield when it received lower precipitation values than what the network used for training.

From Table 4 and Table 6, we observed that Hyper3DNetReg-N5 consistently outperformed Hyper3DNetReg-N3 and Hyper3DNetReg-N1, and, in turn, Hyper3DNetReg-N3 consistently yielded better performance than Hyper3DNetReg-N1. This supports our initial hypothesis that our Hyper3DNetReg network with a two-dimensional output would lead us to generate more accurate predicted yield maps compared to methods that utilize a single output. For example, Figure 7d shows that the map generated by Hyper3DNetReg-N1 is noisier than that generated by Hyper3DNetReg-N3 (Figure 7c), which, at the same time, is noisier than that generated by Hyper3DNetReg-N5 (Figure 7b). The reason is that, if we increase the output window size, we also increase the number of overlapping pixels during the predicted yield map generation (as depicted in Figure 4). Thus, for each point of the field, we obtain multiple estimated yield values and the final estimation is defined as the average value. This helps to obtain smoother prediction maps and to reduce the effect of outlier responses.

## 6. Conclusions

Early crop yield prediction while the crop is still growing allows farmers to better allocate expensive farm inputs that can be applied midway through the growing season. Remote sensing of the spatial variability in crop vigor can allow appropriate spatially variable adjustment of the inputs to maximize crop yield and quality as well as maximization of farmer profit and minimization of environmental impact from inputs.

In this paper, we presented a convolutional neural network architecture called Hyper3DNetReg. Our network tackles the yield prediction problem as a two-dimensional regression task and allows predicting the yield values of a small spatial neighborhood of a field simultaneously. Experimental results showed that, when using training sets with reasonable data representativeness, our model generated the most accurate predicted yield maps on four different fields in comparison to other models that used one-dimensional outputs.

A possible limitation is that the highly non-linear functions learned by our deep learning models prevent humans from interpreting their behavior. Future work will focus on the use of our models for the analysis and understanding of nitrogen-response curves through an explainable machine learning technique based on counterfactual explanations. In addition, we are in the process of extending our models to calculate narrow and accurate prediction intervals automatically to estimate a degree of confidence alongside each prediction. We also plan to evaluate the performance of our Hyper3DNet model on different types of crops such as corn and soybean.

## Figures and Tables

**Table 1:** Number of samples and years of observation for each field.

Field	# Samples 1st Year	# Samples 2nd Year	# Samples 3rd Year	Observed Years
F1	408	316	317	2016, 2018, 2020
G1	484	497	614	2016, 2018, 2020
G2	1014	920	1014	2016, 2019, 2021
G3	290	414	—	2017, 2020

**Table 2:** Splits used to create the training, validation, and test sets.

Field	Split	Training + Validation	Test
F1	A	2016, 2018	2020
	B	2016, 2020	2018
	C	2018, 2020	2016
G1	A	2016, 2018	2020
	B	2016, 2020	2018
	C	2018, 2020	2016
G2	A	2016, 2019	2021
	B	2016, 2021	2019
	C	2019, 2021	2016
G3	A	2017	2020
	B	2020	2017

**Table 3:** Hyper3DNetReg architecture.

Layer Name	Kernel Size	Padding Size	Output Size
Input	—	—	(5, 5, *n*, 1)
Conv3D + ReLU + BN	(3, 3, 3)	(1, 1, 1)	(5, 5, *n*, 32)
Conv3D + ReLU + BN	(3, 3, 3)	(1, 1, 1)	(5, 5, *n*, 32)
CONCAT	—	—	(5, 5, *n*, 64)
Conv3D + ReLU + BN	(3, 3, 3)	(1, 1, 1)	(5, 5, *n*, 32)
CONCAT	—	—	(5, 5, *n*, 96)
Conv3D + ReLU + BN	(3, 3, 3)	(1, 1, 1)	(5, 5, *n*, 32)
CONCAT	—	—	(5, 5, *n*, 128)
Reshape	—	—	(5, 5, 128·n)
Dropout (0.5)	—	—	(5, 5, 128·n)
SepConv2D + ReLU + BN	(3, 3)	(1, 1)	(5, 5, 512)
SepConv2D + ReLU + BN	(3, 3)	(1, 1)	(5, 5, 320)
Dropout (0.5)	—	—	(5, 5, 320)
SepConv2D + ReLU + BN	(3, 3)	(1, 1)	(5, 5, 256)
Dropout (0.5)	—	—	(5, 5, 256)
SepConv2D + ReLU + BN	(3, 3)	(1, 1)	(5, 5, 128)
SepConv2D + ReLU + BN	(3, 3)	(1, 1)	(5, 5, 32)
if N=5 or N=3:			
Conv2D + ReLU	(3, 3)	$\left\{\begin{array}{cc} (1,1), & \mathrm{if}\;N=5\\ (0,0), & \mathrm{elif}\;N=3 \end{array}\right.$	(*N*, *N*, 1)
elif N=1:			
Conv2D + ReLU	(3, 3)	(0, 0)	(3, 3, 1)
Reshape	—	—	(9, 1)
FC	—	—	***N***

**Table 4:** Yield prediction comparison (split A). Best-performing metrics are highlighted in bold. $SSIM3^{*}$ and $SSIM11^{*}$ are SSIM values multiplied by 100.

Field	Metric	Hyper3D NetReg N = 1	Hyper3D NetReg N = 3	Hyper3D NetReg N = 5	AdaBoost. App N = 1	SAE N = 1	3D-CNN N = 1	CNN-LF N = 1	RF N = 1	BMLR N = 1	MLR N = 1
F1	RMSE	13.52	11.88	10.88	12.69	10.93	11.64	**10.73**	15.45	10.95	10.98
	RMedSE	8.94	8.10	7.01	7.74	6.75	7.59	7.42	11.42	6.75	**6.74**
	*r*	0.15	0.47	0.50	0.16	0.27	0.26	0.30	0.37	**0.52**	0.51
	$SSIM3^{*}$	33.58	36.21	**43.2**	39.74	41.66	38.85	40.96	38.96	40.37	40.39
	$SSIM11^{*}$	51.87	56.29	**62.23**	58.82	60.99	57.81	60.15	57.83	59.74	59.8
G1	RMSE	17.39	15.93	**15.19**	16.05	15.66	18.94	16.81	18.36	16.82	16.85
	RMedSE	10.09	10.63	**9.04**	9.92	10.19	11.24	10.70	11.81	10.01	10.06
	*r*	0.29	0.34	**0.43**	0.29	0.33	0.32	0.37	0.26	0.37	0.37
	$SSIM3^{*}$	15.93	15.44	15.95	19.73	**22.21**	22.35	23.15	17.4	24.48	24.51
	$SSIM11^{*}$	34.96	36.45	37.01	40.56	**44.0**	44.17	46.03	37.28	46.58	46.61
G2	RMSE	17.05	19.47	**16.71**	17.02	24.81	24.52	21.1	16.97	27.6	28.09
	RMedSE	11.55	15.72	**12.45**	12.66	37.12	18.20	17.70	14.01	20.31	20.92
	*r*	0.37	0.29	0.55	0.43	0.10	0.10	0.29	**0.60**	0.21	0.19
	$SSIM3^{*}$	5.44	5.52	**6.55**	5.92	1.73	3.3	5.56	8.59	2.03	1.88
	$SSIM11^{*}$	13.96	12.69	**15.38**	14.43	5.59	8.9	13.51	18.97	3.69	3.35
G3	RMSE	19.28	19.11	**16.36**	23.19	17.62	21.44	42.86	18.34	23.9	27.57
	RMedSE	14.51	13.75	**11.26**	16.71	13.32	13.73	14.88	11.98	15.35	19.19
	*r*	0.35	0.54	**0.64**	0.52	0.58	0.31	0.30	0.45	0.56	0.55
	$SSIM3^{*}$	25.36	27.22	**29.49**	25.2	27.81	27.37	23.5	23.83	26.25	23.12
	$SSIM11^{*}$	48.24	51.43	**54.05**	48.32	52.55	47.79	37.78	47.12	45.32	39.61

**Table 5:** Yield prediction comparison (split B). Best-performing metrics are highlighted in bold. $SSIM3^{*}$ and $SSIM11^{*}$ are SSIM values multiplied by 100.

Field	Metric	Hyper3D NetReg N = 1	Hyper3D NetReg N = 3	Hyper3D NetReg N = 5	AdaBoost. App N = 1	SAE N = 1	3D-CNN N = 1	CNN-LF N = 1	RF N = 1	BMLR N = 1	MLR N = 1
F1	RMSE	19.04	18.46	19.34	14.98	21.41	20.43	17.83	19.15	**14.42**	14.94
	RMedSE	14.27	15.18	16.96	9.74	20.43	8.40	9.47	16.73	5.96	**5.80**
	*r*	0.19	0.18	0.21	0.15	0.18	**0.27**	0.26	0.14	0.19	0.19
	$SSIM3^{*}$	26.54	29.12	28.63	30.01	28	**32.14**	29.42	30.34	30.68	30.58
	$SSIM11^{*}$	57.24	60.47	59.98	63.35	57.67	**66.5**	64.68	59.62	66.36	66.28
G1	RMSE	23.82	26.41	27.01	24.34	19.04	33.03	23.24	19.19	**19.62**	20.42
	RMedSE	14.96	17.98	20.53	17.57	12.96	26.88	13.95	**12.92**	13.22	14.01
	*r*	0.35	0.27	0.41	0.41	0.30	0.33	0.19	**0.53**	0.43	0.43
	$SSIM3^{*}$	16.38	18.39	14.64	22.51	25.47	24.97	23.67	**28.88**	26.72	26.64
	$SSIM11^{*}$	42	41.16	36.88	46.27	52.16	45.13	46.2	**57.19**	54.86	54.72
G2	RMSE	43.42	43.36	46.49	43.6	46.64	44.92	41.22	39.6	35.68	**35.4**
	RMedSE	37.74	36.47	40.04	37.09	40.48	38.18	34.35	32.88	27.71	**27.34**
	*r*	0.17	0.21	0.23	0.03	0.04	**0.28**	0.18	0.24	0.03	0.03
	$SSIM3^{*}$	9.69	8.96	10.01	7.46	6	9.09	9.32	**12.39**	9.74	9.34
	$SSIM11^{*}$	21.33	20.72	20.21	19.06	15.95	19.51	21.6	**25.71**	24.87	24.38
G3	RMSE	21.79	18.51	18.09	21.51	19.39	18.6	21.6	21.97	**17.96**	27.76
	RMedSE	13.53	11.57	11.53	14.55	12.02	**11.08**	14.13	16.35	11.44	22.08
	*r*	0.47	0.49	0.53	0.57	0.57	0.51	0.40	0.47	0.57	**0.58**
	$SSIM3^{*}$	32.09	37.19	41.85	40.65	40.52	35.34	26.88	37.08	**44.17**	39.5
	$SSIM11^{*}$	54.94	60.31	64.63	62.64	62.39	60.47	50.08	60.74	**66.72**	59.6

**Table 6:** Yield prediction comparison (split C). Best-performing metrics are highlighted in bold. $SSIM3^{*}$ and $SSIM11^{*}$ are SSIM values multiplied by 100.

Field	Metric	Hyper3D NetReg N = 1	Hyper3D NetReg N = 3	Hyper3D NetReg N = 5	AdaBoost. App N = 1	SAE N = 1	3D-CNN N = 1	CNN-LF N = 1	RF N = 1	BMLR N = 1	MLR N = 1
F1	RMSE	23.62	20.97	19.39	17.86	16.95	19.85	15.13	13.28	13.43	**13.13**
	RMedSE	21.49	19.02	17.44	13.93	14.48	17.35	11.85	9.85	10.57	**10.21**
	*r*	0.25	0.32	**0.34**	0.27	0.24	0.28	0.24	0.29	0.27	0.27
	$SSIM3^{*}$	19.27	19.72	22.27	20.89	20.26	19.64	19.86	21.97	**22.0**	21.51
	$SSIM11^{*}$	42.15	46.16	49.2	45.96	47.77	45.04	46.61	**50.69**	50.6	50.11
G1	RMSE	16.85	14.31	**12.88**	16.85	14.12	14.88	18.72	16.27	18.13	20.43
	RMedSE	10.82	9.43	**8.57**	11.51	9.82	10.35	12.30	11.51	12.29	13.33
	*r*	0.23	0.24	**0.31**	0.21	0.15	0.26	0.29	0.11	0.12	0.11
	$SSIM3^{*}$	16.17	17.68	**20.01**	17.27	17.11	18.35	16.22	17.75	15.54	14.5
	$SSIM11^{*}$	37	43.91	**46.91**	39.62	42.47	42.25	35.64	40.86	36.2	31.21
G2	RMSE	18.05	17.22	**16.66**	16.17	18.09	16.69	22.83	30.69	16.81	16.84
	RMedSE	11.24	11.78	**10.48**	11.70	12.50	11.56	18.72	23.04	11.67	11.65
	*r*	0.21	0.37	**0.41**	0.05	0.08	0.10	0.38	0.10	0.18	0.18
	$SSIM3^{*}$	4.93	8.64	**7.39**	5.37	2.5	6.18	7.57	4.82	5.72	5.75
	$SSIM11^{*}$	12.86	17.91	**16.98**	14.05	10.11	14.18	16.95	12.93	15.16	15.2

**Table 7:** Average annual precipitation per field (in mm).

Split	1st Year	2nd Year	3rd Year
F1	86	130	101
G1	82.35	199.5	66.1
G2	78.9	92.6	60.8
G3	66	105.5	—

## Data Availability

We do not have permission to share farmers’ data.
